# *Staphylococcus aureus* Host Cell Invasion and Virulence in Sepsis Is Facilitated by the Multiple Repeats within FnBPA

**DOI:** 10.1371/journal.ppat.1000964

**Published:** 2010-06-24

**Authors:** Andrew M. Edwards, Jennifer R. Potts, Elisabet Josefsson, Ruth C. Massey

**Affiliations:** 1 Department of Biology and Biochemistry, University of Bath, Bath, United Kingdom; 2 Department of Biology and Department of Chemistry, University of York, York, United Kingdom; 3 Department of Rheumatology and Inflammation Research, University of Gothenburg, Gothenburg, Sweden; Dartmouth Medical School, United States of America

## Abstract

Entry of *Staphylococcus aureus* into the bloodstream can lead to metastatic abscess formation and infective endocarditis. Crucial to the development of both these conditions is the interaction of *S. aureus* with endothelial cells. *In vivo* and *in vitro* studies have shown that the staphylococcal invasin FnBPA triggers bacterial invasion of endothelial cells via a process that involves fibronectin (Fn) bridging to α_5_β_1_ integrins. The Fn-binding region of FnBPA usually contains 11 non-identical repeats (FnBRs) with differing affinities for Fn, which facilitate the binding of multiple Fn molecules and may promote integrin clustering. We thus hypothesized that multiple repeats are necessary to trigger the invasion of endothelial cells by *S. aureus*. To test this we constructed variants of *fnb*A containing various combinations of FnBRs. *In vitro* assays revealed that endothelial cell invasion can be facilitated by a single high-affinity, but not low-affinity FnBR. Studies using a nisin-inducible system that controlled surface expression of FnBPA revealed that variants encoding fewer FnBRs required higher levels of surface expression to mediate invasion. High expression levels of FnBPA bearing a single low affinity FnBR bound Fn but did not invade, suggesting that FnBPA affinity for Fn is crucial for triggering internalization. In addition, multiple FnBRs increased the speed of internalization, as did higher expression levels of FnBPA, without altering the uptake mechanism. The relevance of these findings to pathogenesis was demonstrated using a murine sepsis model, which showed that multiple FnBRs were required for virulence. In conclusion, multiple FnBRs within FnBPA facilitate efficient Fn adhesion, trigger rapid bacterial uptake and are required for pathogenesis.

## Author Summary

*Staphylococcus aureus* is a frequent cause of bacteremia and sepsis. Adhesion to and invasion of endothelial cells lining blood vessels by *S. aureus* can lead to colonization of the heart valves and/or dissemination into surrounding tissues and the establishment of secondary (metastatic) infections. Uptake by endothelial cells is triggered by the interaction of fibronectin-binding protein A (FnBPA) with host cell receptors called integrins via a fibronectin (Fn) bridge. FnBPA contains 11 non-identical repeats (FnBRs) that mediate binding to Fn. Previous work has shown that partial deletions in the FnBR region do not significantly affect binding to Fn or invasion of host cells *in vitro*. It was therefore unclear why FnBPA contains so many repeats. This work demonstrates that the number of repeats affects the amount of surface-exposed FnBPA required to trigger invasion, as well as affecting the speed of uptake. As such, the presence of multiple repeats within FnBPA facilitates adhesion and invasion even at low surface expression levels. Experiments using a murine model of sepsis demonstrate that these differences have a significant impact on virulence; *S. aureus* expressing FnBPA variants with no or few FnBRs are significantly less virulent compared to FnBPA with the full complement of repeats.

## Introduction

*Staphylococcus aureus* is a major human pathogen and the continuous emergence and spread of antibiotic resistant strains (e.g. MRSA, VRSA) mean treatment options are often severely limited [1], [2]. Despite its normal role as a commensal organism living asymptomatically in the nasal cavities of a large proportion of the human population [3], *S. aureus* is also responsible for a raft of different infections that range in both anatomical site and severity. These infections are facilitated by a vast array of different virulence factors such as adhesins, invasins, toxins and modulins, which not only enable evasion of host immune responses [4], [5], but also contribute to colonization, dissemination, tissue damage and transmission [1].

Whilst some infections are superficial and self-limiting, *S. aureus* is also responsible for serious invasive diseases. Indeed, *S. aureus* is a leading cause of sepsis and infective endocarditis [1], [6]–[8]. Colonization of the heart and subsequent formation of vegetations involves a number of complex interactions [9]–[11]. Animal studies have shown that staphylococcal fibronectin-binding protein A (FnBPA) is able to support the colonization of heart valves by otherwise non-pathogenic *Lactococcus lactis*, as well as promote dissemination into the spleen [12], [13]. In addition, FnBPs were significantly associated with systemic inflammation, severe weight loss and mortality in a murine sepsis model [14]. The role of FnBPA in virulence is supported by *in vitro* work showing that *L. lactis* expressing FnBPA is able to activate endothelial cells, inducing inflammatory and pro-coagulant responses [15], [16]. In addition to binding fibronectin (Fn) and fibrinogen, FnBPA promotes attachment to the endothelium and triggers the uptake of *S. aureus* by endothelial cells, which is believed to facilitate bacterial persistence and the establishment of secondary (metastatic) infections [17]–[21].

In common with several other bacterial pathogens, *S. aureus* invades endothelial cells via cell surface integrins [22]–[24]. The bacterium binds Fn, which is attached to the endothelial cell by α_5_β_1_ integrins [18], [19]. This triggers bacterial uptake via a host-cell driven process involving actin remodelling, focal adhesion kinase and Src family kinases [25], [26]. *In vitro* studies suggest that Fn on the surface of endothelial cells is sufficient for *S. aureus*-integrin bridging and thus binding of exogenous Fn (e.g. from plasma) is not required [19], [26].

In addition to FnBPA, many strains express a second, related, Fn-binding protein (FnBPB). Indeed, the majority of clinical isolates screened encode both (77%), whilst the remainder encode just FnBPA (22%) or, rarely, just FnBPB (1%) [27]. Both proteins have N-terminal domains that bind fibrinogen and elastin [28], [29] followed by a region containing 11 (FnBPA) or 10 (FnBPB) non-identical repeats that are responsible for binding F1 modules in the N-terminal domain of Fn [30], [31]. Studies using synthesized peptides have demonstrated that some FnBRs (1, 4, 5, 9, 10 and 11) have a high affinity for Fn, whilst the others have a much lower affinity [30], [31]. The repeat region is intrinsically disordered, allowing the relatively small FnBRs (27–39 aa) to bind the much larger Fn molecule with high affinity [31]. It has been hypothesized that this arrangement enables a single FnBPA protein to bind multiple Fn molecules, leading to integrin clustering and bacterial uptake [30], [32], [33]. Previous work, using an old organizational scheme for FnBPA based solely on amino acid sequence similarity demonstrated that partial deletions in the repeat region did not detectably diminish the ability of bacteria to bind Fn or invade endothelial cells [13], [19]. However, because it is difficult to relate this work to the new domain organization based on structural data [30], [31], it is not clear how many FnBRs are necessary for, or how the affinity of a given repeat for Fn affects invasion of host cells. Furthermore, it is unknown how the bacterium benefits from such apparent redundancy. This study addresses these questions by using bacteria expressing FnBPA variants containing various combinations of FnBRs to dissect their role in Fn-binding, the invasion of endothelial cells and virulence in a murine sepsis model.

## Results

### Construction of FnBPA variants

**Figure 1:**
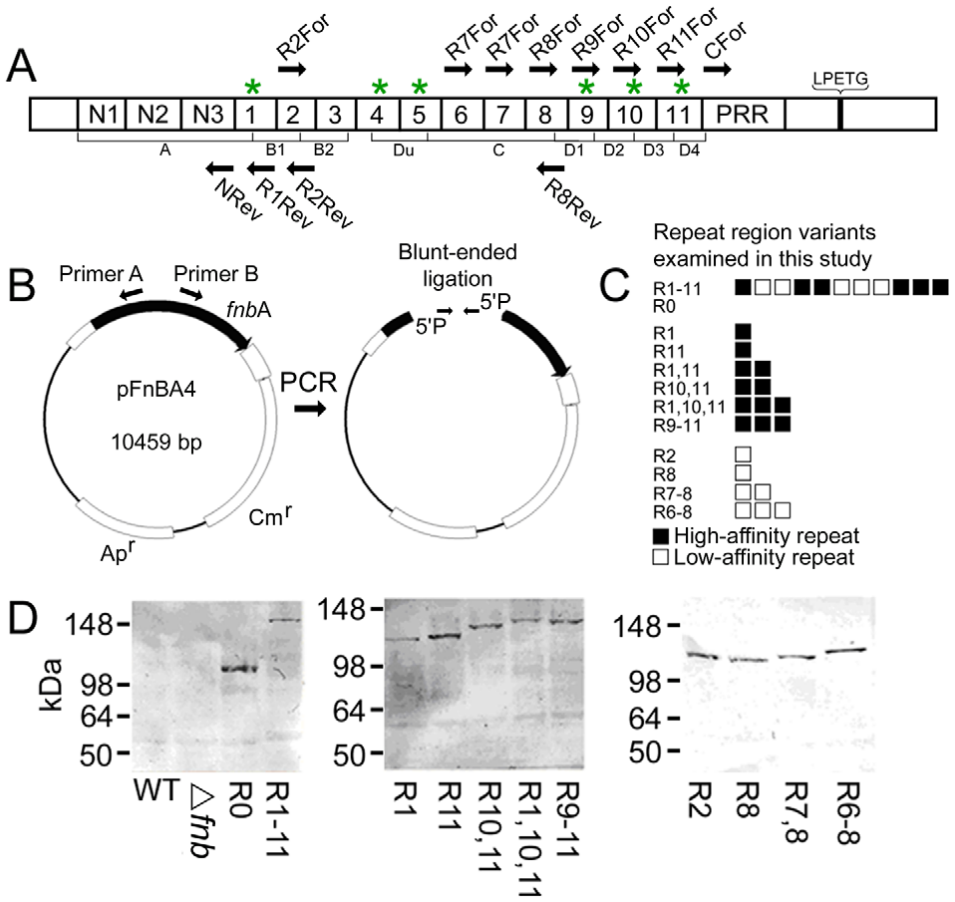
Construction of FnBPA variants containing various repeats. A. Diagrammatic representation of FnBPA, illustrating the relative positions of the N-terminal signal sequence (SS), N-terminal sub-domains (N1, N2 and N3) forming the A domain, 11 Fn-binding repeats (numbered 1–11; those with high affinity for Fn are indicated with asterices), proline-rich region (PRR) and the sortase A recognition sequence LPXTG. The FnBPA variants described in this report differ solely in the composition of the Fn-binding repeat region. Thus FnBPR0 refers to FnBPA without any repeats, FnBPR1 contains only repeat 1 whilst FnBPR1-11 encodes the entire *fnb*A gene etc. The old organizational scheme is shown below the new one. B. Construction strategy used to produce *fnb*A variants containing various combinations of repeats. PCR primers with 5′ phosphate were employed to amplify the entire pFNBA4 plasmid, minus the repeats to be excluded. The blunt ended phosphorylated DNA could then be self-ligated using T4 DNA ligase forming a plasmid containing the modified *fnb*A gene. C. Diagrammatic representation of the composition of the Fn-binding domains of FnBPA variants used in this study. Each square represents a single repeat. Filled squares and open squares represent high- and low-affinity repeats respectively. D. Western immunoblots of surface proteins from *S. aureus* expressing various FnBPA constructs. FnBPA variants were detected using an antibody that recognizes the N-terminal domain that is common to all the constructs used in this study. Blots are labelled according to the repeats present in each of the FnBPA variants expressed.

FnBPA contains 11 non-identical repeats with either high or low affinity for fibronectin (Fig. 1A). FnBPA variants were generated using a technique based on the circular PCR approach described by Massey et al. [13] (Fig. 1A, B and Table 1). Primers were modified to include 5′ phosphate, which facilitated simple blunt-ended ligation of PCR products to form a plasmid encoding *fnb*A variants lacking DNA encoding various repeats (Fig. 1B). Certain constructs were themselves used as templates to produce further *fnb*A variants (Table 1). Previous work (using the old domain organizational scheme (Fig. 1A)), suggested that only a few repeats were needed to confer binding to fibronectin and invasion of endothelial cells [13], [19] and we therefore produced FnBPA variants containing various combinations of 1–3 repeats. As such, 3 different types of construct were produced; those containing a mixture of high- and low-affinity repeats, those containing just high-affinity repeats and those containing just low-affinity repeats (Fig. 1C). Western immunoblots confirmed expression of each of the constructs, which appeared as bands of the expected size (Fig. 1D). DNA sequencing ensured the integrity of each construct (data not shown).

**Table 1:** PCR primer sequences, templates and primer combinations used to make *fnb*A variants.

Template	Primer A	Sequence	Primer B	Sequence	Product
pFnBA4	C For	CCAATCGTGCCACCAACGCC	N Rev	ATTTTTCTCATTTCCGTTCGC	pFnBPR0
	C For	CCAATCGTGCCACCAACGCC	R1 Rev	TGATGAATCATATTCCTCTTCAAC	pFnBPR1
	R9 For	AAATATGAACAAGGTGGCAATATCG	N Rev	ATTTTTCTCATTTCCGTTCGC	pFnBPR9–11
	R10 For	AAGTATGAACATGGCGGTAAC	N Rev	ATTTTTCTCATTTCCGTTCGC	pFnBPR10,11
	R11 For	AGTTATCAATTCGGTGGACAC	N Rev	ATTTTTCTCATTTCCGTTCGC	pFnBPR11
	R10 For	AAGTATGAACATGGCGGTAAC	R1 Rev	TGATGAATCATATTCCTCTTCAAC	pFnBPR1,10,11
	C For	CCAATCGTGCCACCAACGCC	R2 Rev	TGATGAATCCGTTTCTTCTATTGTTTC	pFnE1,2
	C For	CCAATCGTGCCACCAACGCC	R8 Rev	CACGTTGATATTAAGAGTGAATTAGG	pFnR1–8
pFnBPR1,2	R2 For	ACTCTTGACATTGATTACCACACAG	N Rev	ATTTTTCTCATTTCCGTTCGC	pFnBPR2
pFnBPR1–8	R6 for	GAATATACAACTGAAAGTAATC	N Rev	ATTTTTCTCATTTCCGTTCGC	pFnBPR6–8
pFnBPR6–8	R7 For	AACAATCATCATATTTCTC	N Rev	ATTTTTCTCATTTCCGTTCGC	pFnBPR7,8
pFnBPR6–8	R8 for	CACGTTGATATTAAGAGTG	N Rev	ATTTTTCTCATTTCCGTTCGC	pFnBPR8

### Surface expression levels of FnBPA are equivalent between variants

**Figure 2:**
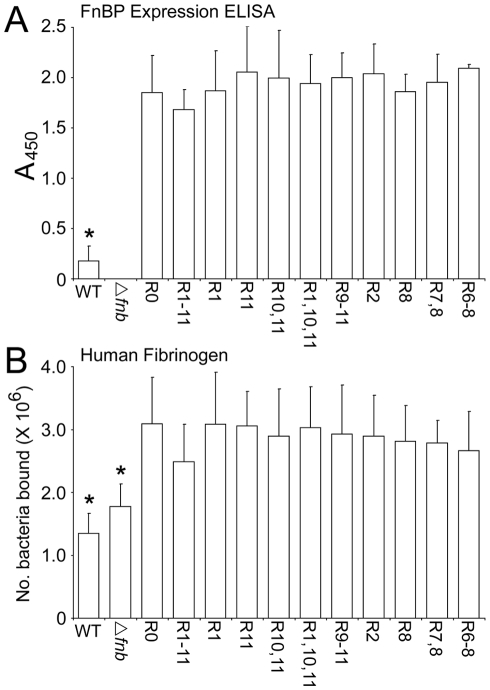
FnBPA variants are expressed at equivalent levels on the surface of *S. aureus* 8325.4. A. Reactivity of anti-N-terminal FnBPA antibodies with *S. aureus* 8325.4 WT and a Δ*fnb* isogenic mutant expressing plasmid-encoded FnBPA variants. An isogenic mutant deficient in FnBPs (Δ*fnb*) was used as a blank to control for interference by protein A. Standard plots of ELISA values against FnBPA can be found in Figure S1. B. Binding of *S. aureus* 8325.4 expressing FnBPA variants to fibrinogen immobilized onto plastic wells. Wild-type (WT) *S. aureus* 8325.4, an isogenic mutant deficient in FnBPs (Δ*fnb*) and the isogenic mutants expressing a range of different FnBPA variants to Fn (1 µg per well) was assessed after incubation at 37°C for 1 h. Binding levels that were significantly different (p = <0.05) from FnBPR0 are indicated (*). Experiments were repeated 4 times in duplicate.

To verify that each of the FnBPA variants were expressed at equivalent concentrations we employed an ELISA approach using antibodies raised to the N-terminal region of FnBPA that is conserved between our variants. Standard plots (Fig. S1) indicated that this would allow us to accurately measure FnBPA expression levels and detect any differences between variants. Because *S. aureus* expresses protein A, we used a *S. aureus* Δ*fnb*A/B (Δ*fnb*) strain (DU5883) as the blank in our ELISA assay, meaning that values relate purely to FnBPA expression levels. As expected from our Western immunoblot data (Fig. 1D), FnBPA expression levels in the 8325.4 wild-type (WT) strain were low (Fig. 2A). These levels were higher amongst the bacteria expressing plasmid-encoded FnBPA, which were equivalent between variants (Fig. 2A).

We also measured FnBPA expression by quantifying adhesion of *S. aureus* expressing each FnBPA variant to immobilized fibrinogen (Fig. 2B). Fibrinogen binding is conferred by the N-terminal region that is constant in all of the variants [28]. Attachment to fibrinogen was maintained in the 8325.4 Δ*fnb* strain due to the presence of fibrinogen-binding ClfA and ClfB proteins. However, binding of *S. aureus* expressing plasmid-encoded FnBPA was significantly enhanced above that of the 8325.4 WT or Δ*fnb* strains, reflecting the higher levels of plasmid-encoded FnBPA, which was equal between variants indicating equivalent expression levels (Fig. 2B).

### A single high-affinity FnBR is sufficient for FnBPA-mediated adhesion to Fn and invasion of endothelial cells

**Figure 3:**
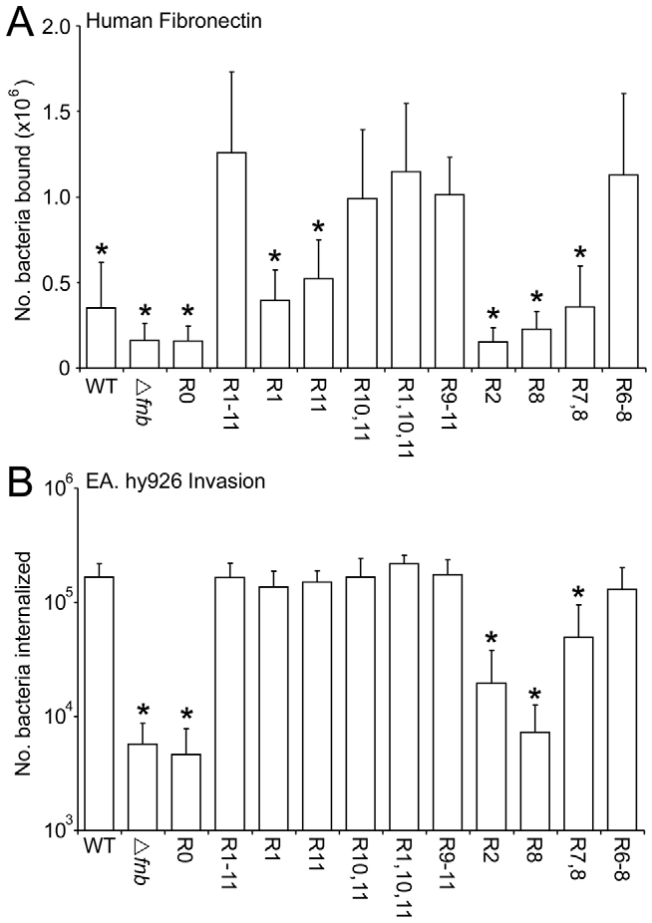
Composition of the Fn-binding repeat region significantly affects adhesion and internalization of *S. aureus* 8325.4. A. Adhesion of *S. aureus* expressing FnBPA variants to Fn immobilized onto plastic wells. Wild-type (WT) *S. aureus* 8325.4, an isogenic mutant deficient in FnBPs (▵*fnb*) and the isogenic mutants expressing a range of different FnBPA variants to Fn (1 µg per well) was assessed after incubation at 37°C for 1 h. Binding levels that were significantly different (p = <0.05) from FnBPR1-11 are indicated (*). Experiments were repeated 4 times in duplicate. B. Uptake of *S. aureus* expressing FnBPA variants by EA. hy926 endothelial cells. The number of intracellular bacteria, including wild-type (WT) *S. aureus* 8325.4, an isogenic mutant deficient in FnBPs (Δ*fnb*) and the isogenic mutants expressing a range of different FnBPA variants, within EA. hy926 cells was determined after incubation at 37°C in 5% CO_2_ for 1 h using the gentamicin protection assay (see Materials and Methods section). In each case experiments were repeated 4 times in duplicate wells. Invasion levels that were significantly different (p = <0.05) from FnBPR1-11 are indicated (*).

Previous work using synthesized peptides [31] determined that some FnBRs have a high affinity for Fn, whilst others bind more weakly (Fig. 1A). To examine how the composition of the Fn-binding region of FnBPA affects both adhesion to Fn and endothelial cell invasion, various combinations of FnBPA repeats were expressed on the surface of a *S. aureus* 8325.4 mutant strain lacking both FnBPA and FnBPB [34]. In keeping with previous work [19], [34], *S. aureus* lacking FnBPs exhibited significantly reduced binding to Fn (Fig. 3A) and invasion of endothelial cells (Fig. 3B; Fig. S2). *S. aureus* expressing FnBPA lacking the entire Fn-binding repeat region (FnBPR0) did not adhere to Fn or invade endothelial cells above the levels of the FnBP-deficient mutant (Fig. 3A, B). Expression of FnBPA from the plasmid was higher than that of WT bacteria (Fig. 2A, B) and accordingly *S. aureus* FnBPR1-11 bound Fn more strongly than WT 8325.4 (Fig. 3A). Bacteria expressing FnBPA variants with increasing numbers of high-affinity repeats bound Fn incrementally, where three repeats were sufficient to confer the same level of binding to Fn as *S. aureus* expressing the full length protein (or for consistency in nomenclature here FnBPR1-11) (Fig. 3A). Despite binding Fn at a lower level, a single high-affinity repeat (FnBPR1 or FnBPR11) was sufficient to confer equivalent invasion of endothelial cells when compared with the full length protein. A single low-affinity repeat (FnBPR2 or FnBPR8) did not confer any binding to Fn above that observed for the knockout strain, but the addition of an extra low-affinity repeat (FnBPR7,8) did increase this, albeit at a significantly lower level when compared with the high-affinity repeats (Fig. 3A). Three low-affinity repeats (FnBPR6–8) were needed before adhesion to Fn or invasion of endothelial cells was equivalent to that conferred by the full length protein (Fig. 3A, B).

**Figure 4:**
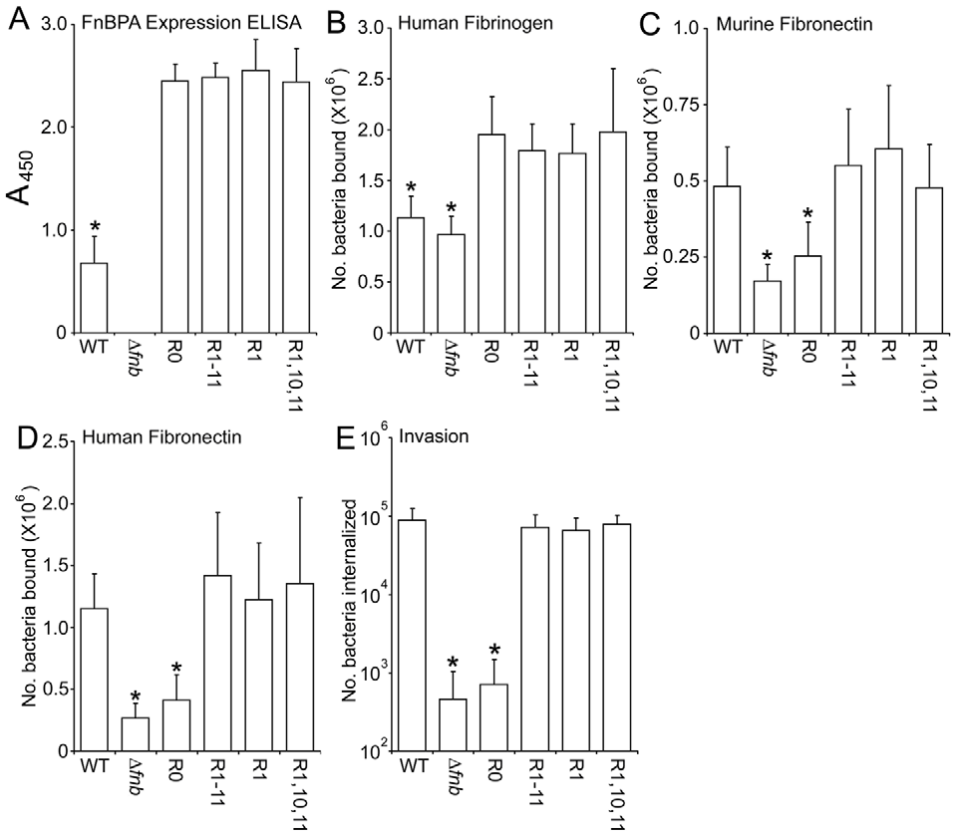
Expression levels and phenotype of *S. aureus* LS-1 expressing FnBPA variants. Reactivity of anti-N-terminal FnBPA antibodies with *S. aureus* LS-1 WT and a Δ*fnb* isogenic mutant expressing plasmid-encoded FnBPA variants. An isogenic mutant deficient in FnBPs (Δ*fnb*) was used as a blank to control for interference by protein A. Standard plots of ELISA values against FnBPA can be found in Figure S1. B. Binding to human fibrinogen (1 µg per well) by *S. aureus* LS-1 wild-type (WT), Δ*fnb* and Δ*fnb* expressing FnBPA variants. Binding was assessed after incubation at 37°C for 1 h. Binding levels that were significantly different (p = <0.05) from FnBPR0 are indicated (*). C. Attachment to murine Fn (1 µg per well) by the same strains described above. Binding levels that were significantly different (p = <0.05) from WT are indicated (*). D. Attachment to human Fn (1 µg per well) by the same strains. Binding levels that were significantly different (p = <0.05) from WT are indicated (*). All binding assays were repeated 4 times in duplicate wells. E. Uptake by EA. hy926 endothelial cells by the same strains. The number of intracellular bacteria was determined after incubation at 37°C in 5% CO_2_ for 1 h using the gentamicin protection assay (see Experimental procedures section). In each case experiments were repeated 4 times in duplicate wells. Invasion levels that were significantly different (p = <0.05) from WT are indicated (*).

Selected constructs were also expressed in the mouse-derived *S. aureus* strain LS-1. Initial experiments were performed to assess the expression levels of each of the constructs by ELISA using anti-FnBPA antibodies (Fig. 4A) and fibrinogen binding (Fig. 4B), as described above for 8325.4. As for 8325.4, LS-1 WT expressed FnBPA at lower levels than the plasmid-encoded gene. Expression levels were equivalent between variants (Fig. 4A). LS-1 WT and FnBPA-defective strains both bound fibrinogen (due to the presence of fibrinogen-binding proteins ClfA and ClfB [14]) but LS-1 strains expressing each of the FnBPA variants bound in 2-fold greater numbers (Fig. 4B), reflecting the higher levels of FnBPA expression that occurs when the gene is located on the plasmid. There were no differences in binding levels between plasmid-encoded FnBPA variants (Fig. 4B). Binding to either human or murine Fn was dependant on the presence of at least 1 FnBR (Fig. 4C, D). Similarly, invasion of EA. hy926 cells was dependent on the presence of at least a single high-affinity FnBR (Fig. 4E).

### A nisin-inducible system facilitates controlled surface expression of FnBPA variants

**Figure 5:**
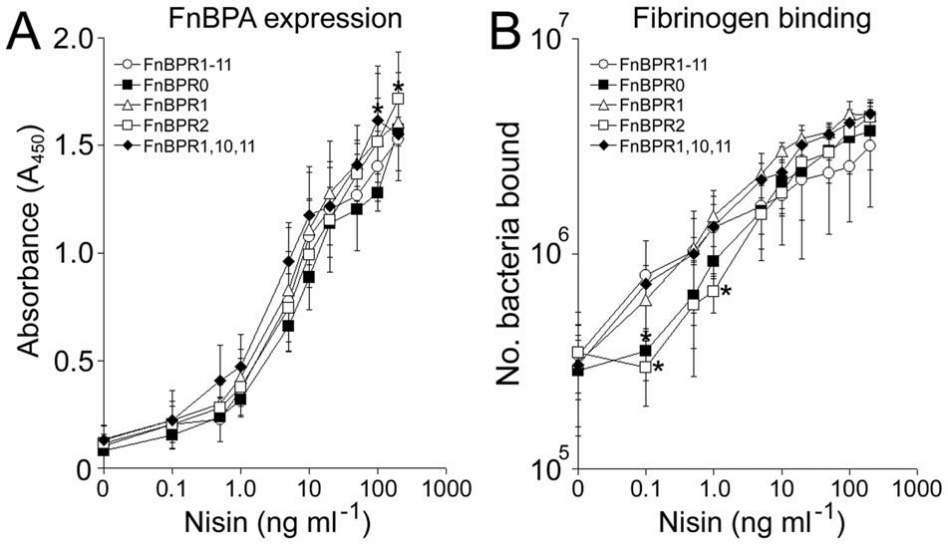
Nisin controlled expression of FnBPA in *L. lactis* is consistent between variants. A. Expression of FnBPA variants (as assessed by A_450_ readings) by *L. lactis* FnBPR1-11 (open circles), FnBPR0 (closed squares), FnBPR1 (triangle), FnBPR2 (open squares) and FnBPR1,10,11 (diamonds) grown to stationary phase (16 h) in the presence of increasing concentrations of nisin (0–200 ng ml^−1^). B. Adhesion of *L. lactis* FnBPR1-11 (open circles), FnBPR0 (closed squares), FnBPR1 (triangle), FnBPR2 (open squares) and FnBPR1,10,11 (diamonds) to immobilized fibrinogen after growth to stationary phase in the presence of increasing concentrations of nisin (0–200 ng ml^−1^). Values that were significantly different (p = <0.05) from FnBPR1-11 are indicated (*). Experiments were repeated 3 times in duplicate.

Although FnBPA variants expressing a single or very few high-affinity repeats were able to mediate adhesion and invasion at the same level as full length FnBPA, we considered the possibility that the relatively high levels of protein expression of the constructs (Fig. 2A, B) might mask important differences between full length FnBPA and variant forms. As such we chose the following constructs for further study; FnBPR1-11 (full-length protein), FnBPR1 (single high-affinity repeat), FnBPR2 (single low-affinity repeat) and FnBPR1, 10, 11 (3 high-affinity repeats and binds and invades equivalently to FnBPR1-11). FnBPR0 was included as a control as it wouldn't be expected to bind or invade at any expression level. To assess how selected FnBPA variants might affect adhesion and internalization at lower expression levels an inducible expression system was employed [35]. This and similar systems have been used previously to vary expression levels of several Gram-positive proteins on the surface of *L. lactis* [35]–[37]. Surface expression of FnBPA variants was quantified by ELISA using antibodies to the A domain of FnBPA (which is common to all variants). Analysis of *L. lactis* expressing FnBPR1-11 grown in a range of nisin concentrations (0–200 ng ml^−1^) revealed that, in keeping with previous reports [35], [36], low-level expression occurred in the absence of nisin (Fig. 5A). A dose-dependent increase in FnBPR1-11 expression was seen with increasing concentrations of nisin up to 200 ng ml^−1^ (Fig. 5A), beyond which bacterial growth was inhibited (data not shown). Expression levels of each of the FnBPA variants were equivalent when identical nisin concentrations were used (Fig. 5A). In addition to the ELISA we employed a fibrinogen-binding assay to assess FnBPA function as the A domain, which is constant in all our constructs, is known to bind fibrinogen (Fig. 5B). In the absence of nisin *L. lactis* expressing each FnBPA variant bound fibrinogen at very low levels, which increased in a dose-dependent manner and were equal at nisin concentrations >10 ng ml^−1^ (Fig. 5B). Adhesion to fibrinogen at lower surface expression levels (<10 ng ml^−1^) was variable and, in keeping with recent work [13], suggested that FnBR1 is required for optimal binding to this ligand.

### Multiple FnBRs within FnBPA support Fn adhesion and trigger bacterial internalization at low levels of surface expression

**Figure 6:**
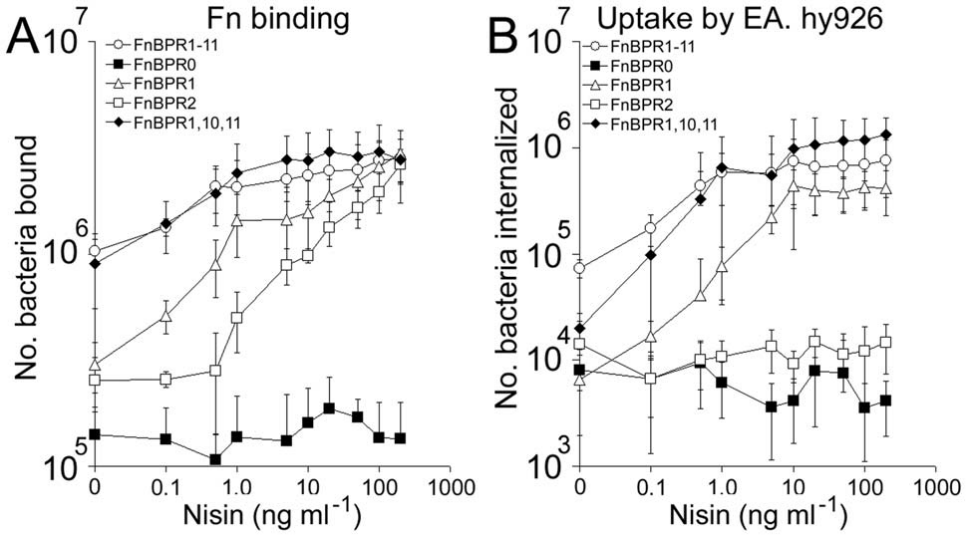
Full-length FnBPA supports adhesion and invasion even at low surface density. A. *L. lactis* FnBPR1-11 (open circles), FnBPR0 (squares), FnBPR1 (triangles), FnBPR2 (closed circles) and FnBPR1,10,11 (diamonds) were grown to stationary phase in the presence of various concentrations of nisin and assessed for their ability to bind Fn. Experiments were repeated 4 times in duplicate. B. *L. lactis* strains were cultured as described in (A) and their abilities to trigger uptake by EA. hy926 cells measured by the gentamicin protection assay. Experiments were repeated 4 times in duplicate.

The abilities of five FnBPA variants to adhere to Fn and to trigger bacterial internalization were compared over a range of expression levels. In the absence of nisin, *L. lactis* expressing FnBPR1-11 adhered strongly to Fn (Fig. 6A). Increasing FnBPR1-11 expression (0–10 ng ml^−1^ nisin) promoted adhesion 2.5-fold before reaching saturation (Fig. 6A). These results mirrored those seen for bacterial internalization, with high levels occurring at basal (absence of nisin) FnBPA expression levels, which increased steadily (>10-fold) before reaching a plateau, also at 10 ng ml^−1^ nisin (Fig. 6B). By contrast, binding to Fn or invasion of endothelial cells by *L. lactis* expressing FnBPR0 (no Fn-binding repeats) was not enhanced at any level of expression (Fig. 6). At basal expression levels *L. lactis* expressing FnBPR1 (containing a single high-affinity repeat) bound to Fn at a low level and was not internalized (relative to FnBPR0, Fig. 6). However, both adhesion and invasion levels increased dramatically (20-fold and 63–fold respectively) with increasing levels of expression, eventually reaching similar levels to that of FnBPR1-11 at saturation (Fig. 6). Adhesion of *L. lactis* expressing FnBPR2 (containing a single low-affinity repeat) to Fn occurred only at levels of induction >1 ng ml^−1^ nisin and increased in a dose-dependent manner, eventually reaching levels similar to that supported by FnBPR1 at 100 ng ml^−1^ nisin (Fig. 6A). However, FnBPR2 did not trigger bacterial internalization at any expression level (Fig. 6B).

The adhesion profile of *L. lactis* expressing FnBPR1,10,11 (containing 3 high-affinity repeats) was very similar to that of FnBPR1-11 (Fig. 6A). At the basal-level of expression *L. lactis* FnBR1,10,11 triggered invasion at levels 3.5-fold lower than full length FnBPR1-11 but 3-fold higher than FnBPR1. Invasion levels increased with increasing nisin concentration, reaching the same level as full length FnBPR1-11 at 10 ng ml^−1^ (Fig. 6B).

Together these findings suggest that although a single high affinity repeat can confer wild-type levels of adhesion and invasion, its expression needs to reach a high density before it can do so. The expression of multiple repeats within a single molecule allows invasion of endothelial cells at low FnBPA surface density.

### The rate of invasion of endothelial cells is directly associated with the density of binding repeats expressed on the surface of bacteria

**Figure 7:**
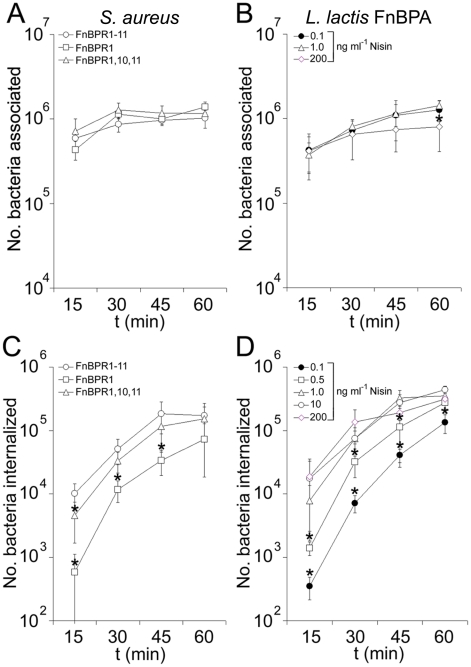
FnBPA composition and expression level modulate the rate of bacterial internalization. A. Total number of *S. aureus* 8325.4 expressing FnBPR1-11 (circles), FnBPR1 (squares) and FnBPR1,10,11 (triangles) associated (adherent and internalized) with endothelial cells at various time points. Values that were significantly different (p = <0.05) from FnBPR1-11 are indicated (*). Experiments were repeated 3 times in duplicate. B. Total number of *L. lactis* expressing FnBPA associated with endothelial cells at various time points after growth in the presence of nisin at 0.1 ng ml^−1^ (closed circles), 1.0 ng ml^−1^ (triangles) or 200 ng ml^−1^ (diamonds). Values that were significantly different (p = <0.05) from *L. lactis* FnBPR1-11 grown in 0.1 ng ml^−1^ nisin are indicated (*). Experiments were repeated 3 times in duplicate. C. Internalization of *S. aureus* FnBPR1-11 (circles), FnBPR1 (squares) and FnBPR1,10,11 (triangles) by EA. hy926 cells over time. Values that were significantly different (p = <0.05) from FnBPR1-11 are indicated (*). Experiments were repeated 3 times in duplicate. D. Internalization of *L. lactis* FnBPR1-11 grown in the presence of nisin at 0.1 ng ml^−1^ (closed circles), 0.5 ng ml^−1^ (squares), 1.0 ng ml^−1^ (triangles), 10 ng ml^−1^ (open circles) and 200 ng ml^−1^ (diamonds) by EA. hy926 cells over time. Values that were significantly different (p = <0.05) from *L. lactis* FnBPR1-11 grown in 200 ng ml^−1^ nisin are indicated (*). Experiments were repeated 3 times in duplicate.

Fn-binding proteins of pathogenic bacteria are believed to cause clustering of cell-surface integrins, leading to cell signalling events that trigger uptake of the bacterium [24]. We hypothesized that multiple FnBRs might trigger uptake in a more efficient manner than a single repeat, leading to an increased rate of bacterial internalization. To assess this we determined the number of adherent and internalized bacteria at various time points from 15 to 60 minutes post-incubation with endothelial cells. Figures 7A and 7B show that adhesion of FnBPA-expressing bacteria to the endothelial cell surfaces was consistent between variants over time. Indeed, adhesion to this cell line does not appear to depend on fibronectin binding since *S. aureus* FnBPR0 binds as efficiently as FnBPR1-11 (data not shown). *S. aureus* expressing full length FnBPR1-11 showed a continual increase in the number of internalized bacteria over this time period, reaching a plateau at 45–60 minutes (Fig. 7C). *S. aureus* expressing only a single repeat (FnBPR1) had approximately 20-fold fewer internalized bacteria after 15 minutes compared with the full length protein but this difference became less pronounced with increasing incubation time, and there was no significant difference in the number of internalized bacteria after 60 minutes (Fig. 7C). *S. aureus* expressing three high affinity repeats invaded more quickly than those expressing a single repeat but less quickly that those expressing all 11 repeats, supporting the hypothesis that multiple repeats increase the rate at which *S. aureus* can invade cells. These results were verified by examining invasion mediated by full length FnBPA on the surface of *L. lactis* at varying densities (Fig. 7D). After 60 minutes' incubation of bacteria and endothelial cells *L. lactis* expressing all densities of FnBPA had invaded at similar levels, but at 15 and 30 minutes incubation a clear association between surface density and bacterial uptake could be seen (Fig. 7D).

### Uptake mechanisms do not differ with varying densities of binding repeats

**Figure 8:**
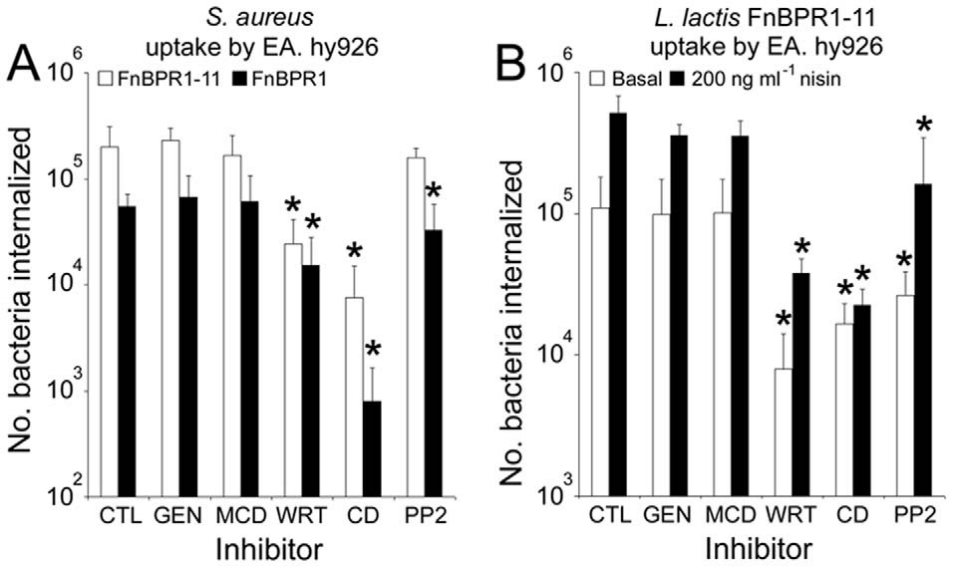
The mechanism of bacterial uptake is unaffected by FnBPA composition or expression level. The number of bacteria taken up by untreated cells (CTL) or those pre-treated with genistein (GEN), methyl-β-cyclodextrin (MCD), wortmannin (WRT), cytochalasin D (CD) or PP2 were measured using the gentamicin protection assay. A. EA. hy926 uptake of *S. aureus* expressing either full length FnBPA (FnBPR1-11, open bars) or FnBPA containing just repeat 1 (FnBPR1, closed bars) after treatment with inhibitors. B. Internalization of *L. lactis* FnBPR1-11 expressing either low levels of FnBPR1-11 (absence of nisin, open bars) or high levels (200 ng ml^−1^ nisin, closed bars) into cells treated with the same inhibitors used in panel A. Graphs represent the mean of 3 independent experiments performed in duplicate with error bars indicating the standard deviation of the mean. Conditions that significantly (p = <0.05) inhibited internalization are indicated (*).

Although triggered by FnBPA, staphylococcal entry into endothelial cells involves host cell processes [18], [20]. There appear to be two major mechanisms of invasion of human cells by pathogenic bacteria, the first involving integrin-triggered remodelling of actin and the second making use of host cell structures known as caveolae [24]. In keeping with previous reports [24]–[26], the invasion of the cells used in this study by *S. aureus* appears to occur via an actin remodelling mechanism (Fig. S2). As the rate of bacterial internalization was affected by FnBPA composition and surface density, we hypothesized that this variation might reflect the triggering of different cellular uptake mechanisms. To examine this we investigated the mode of entry of *S. aureus* expressing FnBPR1-11 and FnBPR1, as well as *L. lactis* expressing FnBPR1-11 at high or low surface densities, using inhibitors of cellular processes. Consistent with wild-type *S. aureus* 8325.4 (Fig. S2D), internalization of *S. aureus* expressing either FnBPR1-11 or FnBPR1 was unaffected by genistein or methyl-β-cyclodextrin but was inhibited by Wortmannin and cytochalasin D (Fig. 8A). Although uptake of both *S. aureus* expressing FnBPR1-11 and FnBPR1 was slightly reduced by the Src kinase inhibitor PP2, it was only significant for FnBPR1 (p = <0.05) (Fig. 8A). *L. lactis* expressing FnBPR1-11 was similarly affected, regardless of expression level (Fig. 8B). Internalization of bacteria weakly (absence of nisin) or strongly (200 ng ml^−1^ nisin) expressing FnBPR1-11 was inhibited by wortmannin and cytochalasin D but not genistein or methyl-β-cyclodextrin (Fig. 8B). Inhibition of *L. lactis* uptake by PP2 was much more pronounced than for *S. aureus*, particularly at low expression levels (Fig. 8). These findings verify that the density of Fn-binding repeats does not affect the uptake mechanism exploited by the bacteria.

### Full-length FnBPA facilitates bacterial adhesion to immobilized Fn in the presence of soluble Fn

**Figure 9:**
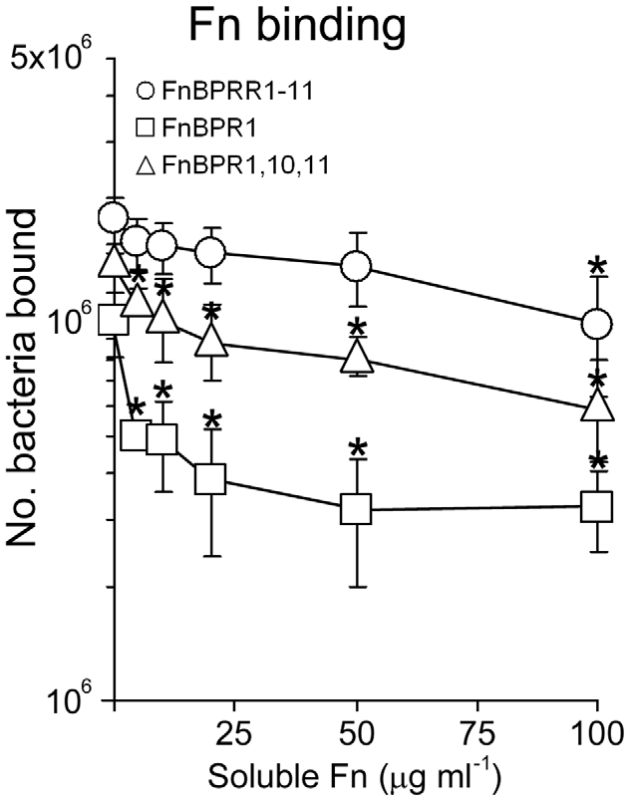
Full length FnBPA facilitates adhesion to immobilized Fn in the presence of soluble Fn. Binding of *S. aureus* expressing various FnBPA variants to Fn immobilized in plastic wells, in the presence of increasing concentrations of fluid-phase Fn. Values that were significantly different (p = <0.05) from binding in the absence of soluble protein (PBS only) are indicated (*). Experiments were repeated 3 times in duplicate.

Blood contains high levels of soluble (plasma) Fn. Although FnBPA has been shown to bind fluid-phase Fn [20], we hypothesized that to bind the endothelium *in vivo S. aureus* must be able to bind preferentially to immobilized (cell bound) Fn. To test this we measured the adhesion of bacteria expressing FnBPA variants to immobilized fibronectin in the presence of increasing concentrations of soluble fibronectin. Adhesion of *S. aureus* expressing FnBPR1-11 to immobilized Fn was unaffected by the presence of soluble Fn except at the highest concentrations used (Fig. 9). By contrast, Fn-binding by *S. aureus* expressing FnBPA variants containing one or three repeats was significantly inhibited at low concentrations of soluble Fn (Fig. 9). This suggests that full-length FnBPA is likely to be significantly more efficient at conferring adhesion to the endothelium under *in vivo* conditions.

### Fibronectin-binding does not affect *S. aureus* survival or modulate TNFα production in whole human blood

**Figure 10:**
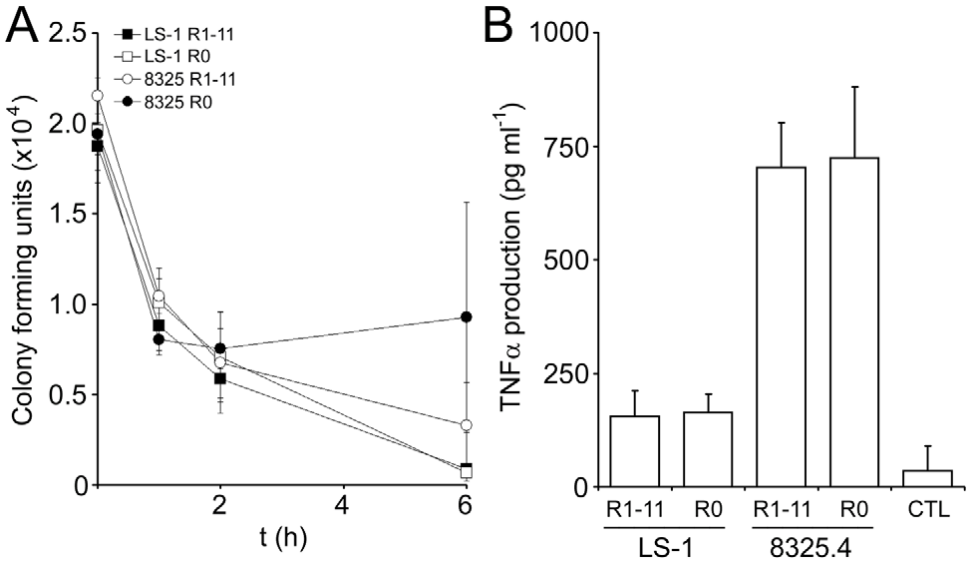
The Fn-binding region of FnBPA does not affect bacterial survival or TNFα production in whole human blood. A. Survival of *S. aureus* 8325.4 expressing FnBPR1-11 (open circles) or FnBPR0 (closed circles) and LS-1 expressing FnBPR1-11 (closed squares) or FnBPR0 (open squares) in whole human blood over time. Aliquots of blood were assayed for CFU at the indicated time points. Experiments were repeated 3 times. B. Production of TNFα by whole human blood in response to *S. aureus* strains 8325.4 and LS-1 expressing either FnBPR1-11 or FnBPR0. Samples were taken after 6 hours and TNFα concentration measured by ELISA. Blood incubated in the absence of bacteria served as a negative control (CTL). Experiments were repeated 3 times.

*S. aureus* has a number of mechanisms that aid evasion of the host immune system including surface adhesins such as Clf and Spa that inhibit phagocytosis [4]. Work with *Streptococcus pyogenes* suggested that the Fn-binding protein PrtF, which is structurally similar to FnBPA, inhibits phagocytosis in a whole blood model [38]. As such, we assessed whether the FnBR region of FnBPA might perform a similar function. Using a similar whole blood model (see Materials and Methods section), we determined the survival of *S. aureus* strains 8325.4 and LS-1 expressing full length FnBPA (FnBPR1-11) or the FnBR-deficient variant FnBPR0. Over a period of up to 6 h there were no significant differences in bacterial CFU between strains expressing FnBPR1-11 or the FnBR-deficient FnBPR0 (Fig. 10A), indicating that fibronectin binding does not affect *S. aureus* survival.

In addition, we assessed whether Fn-binding might affect the inflammatory response of whole blood to *S. aureus* by determining the production of TNFα triggered by each strain. All bacteria examined elicited TNFα expression that was significantly greater than that of whole blood incubated without bacteria (Fig. 10B). Although there was a significant difference between strains, the FnBR-region of FnBPA had no effect on TNFα production by whole blood (Fig. 10B).

### Multiple FnBRs are essential for full virulence in a murine sepsis model

**Figure 11:**
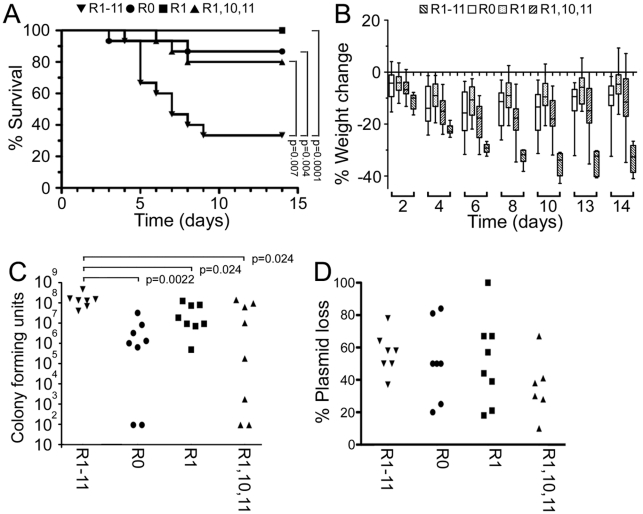
Multiple FnBRs are necessary for full virulence in a murine sepsis model. A. Mice (groups of 15) were inoculated intravenously with approximately 1.1×10^7^ *S. aureus* LS-1 Δ*fnb* expressing FnBPR1-11 (inverted triangles), FnBPR0 (circles), FnBPR1 (squares) or FnBPR1,10,11 (triangles) and survival followed until day 15. B. Weight change in mice infected with strains as described in (A). C. Bacterial load in the kidneys of mice inoculated with bacteria described above. Two independent groups of mice (4 per group) were sacrificed on day 3, their kidneys removed and the number of CFU determined. One mouse in the FnBPR1-11 group died before day three. Two mice each in the FnBPR0 and FnBPR1,10,11 groups did not have detectable numbers of bacteria (<99 CFU). Values that are significantly different (p = <0.05) from *S. aureus* FnBPR1-11 are indicated (*). D. Plasmid loss by *S. aureus* isolated from kidneys 3 days after inoculation. Bacterial colonies used to enumerate bacterial loads in the kidneys were assessed for resistance to chloramphenicol and thus presence or absence of the FnBPA-encoding plasmid.

We hypothesized from our *in vitro* data that bacteria expressing FnBPA variants with multiple repeats would be able to bind to and invade endothelium more efficiently *in vivo* than bacteria expressing FnBPA variants with few or no repeats, causing greater levels of mortality. To test this we employed a murine sepsis model to assess the virulence of *S. aureus* LS-1 (a mouse infective strain) expressing FnBPA variants. *S. aureus* LS-1 Δ*fnb* expressing FnBPR0, FnBPR1, FnBPR1,10,11 or FnBPR1-11 were phenotypically similar to 8325.4 Δ*fnb* expressing the same constructs with respect to Fn-binding and invasion of endothelial cells (Fig. 3). Mice were injected intravenously with one of these four *S. aureus* LS-1 strains and survival and weight loss monitored over 14 days. By day 9, *S. aureus* expressing full length FnBPR1-11 had killed >60% of mice, which was significantly greater than any of the three other strains (p = <0.007, Fig. 11A). Those that survived continued to suffer significant weight loss of approximately 30%, suggesting severe infection (Fig. 11B). By contrast, no significant difference in mortality was observed between *S. aureus* expressing FnBPR0, FnBPR1 or FnBPR1,10,11, which killed up to 20% of the mice (Fig. 11A). The pattern of weight loss was also similar between these three strains, although mice infected with *S. aureus* expressing FnBPR1 and FnBPR1,10,11 showed significantly different weight changes between days 4 and 13 after inoculation (p = 0.02, Fig. 11B).

In addition to measurements of mortality and weight loss we also assessed bacterial numbers (CFU) in the kidneys 3 days after inoculation (Fig. 11C). This time point was chosen because previous experiments (Fig. 11A, B) indicated that it was close to the initiation of mortality (indeed, one mouse in the FnBPR1-11 group died before sacrifice). As expected from the data in Figures 11A and 11B, the kidneys of mice inoculated with *S. aureus* FnBPR1-11 contained significantly more CFU than the kidneys of mice infected with *S. aureus* FnBPR0 (23-fold greater, p = 0.0022). *S. aureus* FnBPR1-11 was also approximately 6-fold more abundant than *S. aureus* expressing either FnBPR1 or FnBPR1,10,11 (p = 0.024). There was no significant difference in the bacterial load between *S. aureus* FnBPR0, FnBPR1 or FnBPR1,10,11. We also used the bacterial colonies from the kidneys to determine loss of the FnBPA-encoding plasmids (Fig. 11D). Plasmid loss was highly variable between animals but was not significantly different between FnBPA variants and did not correlate with bacterial load (data not shown). The difference between *S. aureus* expressing FnBPR0, FnBPR1 or FnBPR1,10,11 when compared with those expressing full length FnBPR1-11 demonstrates that full length FnBPA is required for full pathogenicity in this model, most likely during the early stages of infection.

## Discussion

Bacterial invasion of host cells is a process common to many pathogens. It likely aids evasion of extracellular immune factors and provides a protected niche from antibiotics, and can therefore allow bacteria to cause persistent and recurrent infections [39], [40]. Additionally, bacterial invasion of the endothelium can lead to inflammation, endocarditis and may lead to traversal of blood vessels and subsequent formation of metastatic infections [22]. As such it is crucial that we gain a greater understanding of how this pathogen interacts with the host to identify means of blocking these infections [1].

There is clear and compelling evidence that FnBPA alone is sufficient to trigger *S. aureus* invasion of cells via Fn bridging to α_5_β_1_ integrins [18], [20]. Previous work revealed that staphylococcal attachment to Fn and invasion of host cells is maintained even when substantial portions of the Fn-binding region have been deleted [13], [19]. As such, it was unclear what benefits are conferred by multiple FnBRs. As there is evidence that a single FnBPA molecule is able to bind up to nine Fn molecules [33], we hypothesized that a minimum number of repeats in close proximity would be required to trigger Fn clustering and subsequent integrin clustering, which leads to cell invasion [32], [33]. Furthermore, we hypothesized that the composition of FnBPA variants with respect to high- and low-affinity repeats would affect adhesion and internalization. Here we show that high surface densities of repeats are required for adhesion and internalization *in vitro.* However, it is not important to have multiple repeats within a single FnBPA molecule because high level expression of individual FnBPA variants containing a single high-affinity binding repeat results in invasion at WT levels (Fig. 6). In addition to demonstrating that high surface density of FnBRs promotes cell invasion, we show that speed of uptake is modulated by composition and expression level of FnBPA (Fig. 7).

Although the data presented here suggest that Fn binding is indicative of the potential to trigger invasion, our studies using low-affinity repeats suggest a more complicated situation. The inability of the FnBPA variant containing a single low-affinity repeat (FnBPR2) to trigger internalization at high surface expression levels, despite supporting adhesion to Fn, suggests that invasin-receptor affinity is crucial (Fig. 6). Three tandem low-affinity repeats however support invasion (Fig. 3). It is therefore possible that certain FnBPA-Fn interactions induce a conformational change in the glycoprotein, which triggers integrin signalling and bacterial uptake. *In vivo*, *S. aureus* will not only encounter cell-bound Fn but also the soluble form. Although adhesion of *S. aureus* expressing FnBPR1 and FnBPR1,10,11 to immobilized Fn was significantly inhibited at even low concentrations of soluble Fn, *S. aureus* FnBPR1-11 was only affected at the highest concentration used (Fig. 9). This suggests that the three other high-affinity repeats (4, 5 and 9) and perhaps also the five low-affinity repeats (2, 3, 6, 7 and 8) are important in overcoming the presence of soluble ligand.

Although our data suggest that, in tandem, multiple low-affinity repeats can perform almost as well as single high-affinity repeats, it is not clear what advantage the bacteria gain from maintaining these repeats. The answer may lie in studies of the immune response to FnBPA during staphylococcal infection. Analysis of a panel of antisera from different patients demonstrated that antibodies recognizing the Fn-binding region do not inhibit bacterial binding of Fn [41]. Furthermore, the binding of these antibodies to FnBPA was enhanced in the presence of Fn, presumably due to the creation of ligand-induced binding sites [41]. A more recent study revealed that despite strong antibody recognition of high-affinity repeats when complexed with Fn, there was significantly lower recognition of low-affinity repeats either in the presence or absence of the glycoprotein [31]. As such, the lack of antibody recognition of low-affinity repeats may allow the bacteria to adhere to host tissues even in the presence of anti-FnBPA antibodies.

A role for FnBPA in sepsis has been previously established [14], although it was not clear whether the fibrinogen- and elastin-binding region or the Fn-binding region was important. Using the same model of sepsis we show that the FnBRs are required for virulence (Fig. 11). We also show that increased efficiency of the full length protein (FnBPR1-11) at mediating binding to Fn and cell invasion translates into a difference in virulence. Despite the ability to bind Fn and invade cells *in vitro*, neither *S. aureus* expressing FnBPR1 nor FnBPR1,10,11 were any more virulent than that expressing FnBPR0. The enhanced pathogenesis conferred by multiple FnBRs does not appear to be due to elevated survival in blood or the stimulation of pro-inflammatory TNFα. However, the greater bacterial load in the kidneys of mice infected with *S. aureus* FnBPR1-11, compared with FnBPR0 or FnBPR1, suggests that multiple repeats significantly enhance systemic invasion.

The demonstration of the role of FnBPA in septic death does not rule out the involvement of other staphylococcal factors in the progression of infection. Indeed, a role for clumping factor in sepsis using this model and strain has been shown previously [41] and it is likely that different factors will be required at different stages of the infective process [1]. Experiments examining the loss of FnBPA-encoding plasmids from infecting bacteria suggest that this protein acts early in the infectious process and its retention is not required for the latter stages of infection. Significant plasmid loss at day 3 is unsurprising since the bacterial load for *S. aureus* FnBPR1-11 is 10 times greater than the inoculum, indicating significant replication within this organ. These data indicate that the FnBRs within FnBPA significantly enhance bacterial exit from the blood and colonization of the kidneys soon after entry into the vascular system. This is supported by previous *in vivo* experiments that showed *S. aureus* attachment to the endothelium is dependent on FnBPA and occurs within 5 minutes of inoculation [21]. Once colonization is established, *S. aureus* is likely to employ other virulence factors (e.g. other surface proteins, toxins and proteases), which together with host factors, promote progression to abscess formation [1], leading to weight loss and mortality. However, early extravasation, mediated by FnBPA containing multiple FnBRs, appears to be essential for full virulence.

In summary, the presence of multiple FnBRs within FnBPA results in a high surface density of Fn-binding sites. This triggers a rapid invasion of endothelial cells even at low surface expression levels and enables binding in the presence of soluble ligand. This appears to be particularly relevant to pathogenesis and the development of systemic infection.

## Materials and Methods

### Ethics statement

Approval for experiments using human blood was granted by the Bath Research Ethics Committee (NHS National Research Ethics Services, reference 08/H0101/18). Donors gave informed consent in writing prior to the commencement of any procedures.

Approval for animal experiments was granted by the Göteborg Animal Experimentation Ethics board and their ethical and husbandry guidelines followed for all experiments. NMRI mice were obtained from Charles River (Sulzfeld, Germany) and were maintained in the animal facility of the Department of Rheumatology, University of Göteborg, Sweden. Mice were housed up to 10 animals per cage with a 12 h light-dark cycle, and were fed standard laboratory chow and water ad libitum. The animals were 8 weeks old at the start of the experiments.

The overall condition of each mouse was examined by assessing signs of systemic inflammation, i.e. weight decrease, reduced alertness, and ruffled coat. In keeping with approved husbandry standards, in cases of severe systemic infection, when a mouse was judged too ill to survive another 24 h, it was killed by cervical dislocation and considered dead due to sepsis.

### Bacterial strains and growth conditions

A detailed list of the strains used in this study is presented in Table 2. *S. aureus* strains were cultured for 16 h in Brain-Heart Infusion (BHI) broth at 37°C in air with shaking. *S. aureus* CFU were quantified on Tryptic Soy Agar (TSA) plates incubated overnight at 37°C in air. *L. lactis* strains were cultured in M17 broth (supplemented with 0.5% w/v glucose) for 16 h at 30°C in air (with the appropriate concentration of nisin where necessary). *Escherichia coli* was grown in Luria broth at 37°C with shaking. Where appropriate, bacteria were incubated in the presence of the following antibiotics: ampicillin 100 µg ml^−1^, chloramphenicol 10 µg ml^−1^ and erythromycin 5 µg ml^−1^ (*L. lactis*) or 250 µg ml^−1^ (*E. coli*).

**Table 2:** Bacterial strains and plasmids used in this study.

Species/strain/plasmid	Relevant characteristics	Source/Reference
***E.coli***		
*E. coli* DH5α	Cloning host	This laboratory
(pFnBA4)	Shuttle vector containing the complete *fnb*A gene	[34]
(pMSP7517)	Nisin-inducible vector containing the *prg*B gene of *Enterococcus faecalis*	[35]
*E. coli* K12 ER2925	Cloning host	New England Biolabs
***Staphylococcus aureus***		
*S. aureus* RN4220	Cloning host capable of sustaining shuttle plasmids	[45]
*S. aureus* 8325.4	Wild-type, *fnb*A+ *fnb*B+ NCTC 8325 cured of prophages	[46]
DU5883	*fnb*A- *fnb*B- isogenic mutant of 8325.4	[34]
DU5875	Protein A-deficient isogenic mutant of 8325.4	[47]
DU5883 (pFnBA4) (referred to here as FnBPR1-11)	*fnb* - strain complemented with the plasmid pFnBA4 expressing full-length FnBPA: FnBPR1-11	[34]
pFnBPR0	Expresses FnBPA variant containing no Fn-binding repeats: FnBPR0	This study
pFnBPR1	Expresses FnBPA variant containing Fn-binding repeat 1 only: FnBPR1	This study
pFnBPR11	Expresses FnBPA variant containing Fn-binding repeat 11 only: FnBPR11	This study
pFnBPR10,11	Expresses FnBPA variant containing Fn-binding repeats 10 & 11: FnBPR10& 11	This study
pFnBPR1,10,11	Expresses FnBPA variant containing Fn-binding repeats 1, 10 & 11: FnBPR1, 10, 11	This study
pFnBPR9-11	Expresses FnBPA variant containing Fn-binding repeats 9-11: FnBPR9-11	This study
pFnBPR2	Expresses FnBPA variant containing Fn-binding repeat 2 only: FnBPR2	This study
pFnBPR8	Expresses FnBPA variant containing Fn-binding repeat 8 only: FnBPR8	This study
pFnBPR7,8	Expresses FnBPA variant containing Fn-binding repeats 7 & 8: FnBPR7,8	This study
pFnBPR6-8	Expresses FnBPA variant containing Fn-binding repeats 6-8: FnBPR6-8	This study
*S. aureus* LS-1	Wild-type, *fnb*A+ *fnb*B+ pathogenic in mouse model of infection	[48]
*S. aureus* LS-1 Δ*fnb*	*fnb*A- *fnb*B- isogenic mutant of LS-1	[14]
***Lactococcus lactis***		
*L. lactis* MG1363	Wild-type heterologous expression host (FnBPA/B-)	[19]
pKS80	Empty expression vector	[49]
pRM9	Expresses full length FnBPA of 8325-4	[19]
*L. lactis* NZ9800	*nis*A defective isogenic mutant of NZ9700	[50]
pMFnBPR1-11	Nisin controlled expression of FnBPA containing repeats 1-11: FnBPR1-11	This study
pMFnBPR0	Nisin controlled expression of FnBPA containing no repeats: FnBPR0	This study
pMFnBPR1	Nisin controlled expression of FnBPA containing repeat 1: FnBPR1	This study
pMFnBPR2	Nisin controlled expression of FnBPA containing repeat 2: FnBPR2	This study
pMFnBPR1,10,11	Nisin controlled expression of FnBPA containing repeats 1,10,11: FnBPR1,10,11	This study

### Endothelial cell culture

The cell line EA. hy926, established by the fusion of human umbilical endothelial cells (HUVEC) and the permanent lung epithelial carcinoma cell line A549 [42], was cultured in Dulbecco's Modified Eagle's medium (DMEM; Invitrogen) supplemented with foetal bovine serum (FBS; 10%) and L-glutamine (2 mM) at 37°C and 5% CO_2_. Pooled primary HUVECs were purchased from Lonza (Basel, Switzerland) and cultured in endothelial basal medium supplemented with 2% fetal bovine serum, bovine brain extract (including heparin), human endothelial growth factor, hydrocortisone and GA-1000 (Gentamicin & Amphotericin B) at 37°C and 5% CO_2_ according to manufacturer's instructions (Lonza).

Endothelial cells were cultured in T75 flasks to approximately 95% confluency, liberated with trypsin-EDTA, resuspended in the relevant culture medium and added to 24-well plates containing thermanox glass coverslips [19]. Plates were incubated for 48 h as described above before the coverslips were removed, dip washed in PBS and added to new 24-well plates containing fresh medium and bacteria [19]. In experiments using metabolic inhibitors, these were incubated with the cells 1 h prior to the addition of bacteria and concentrations maintained during the assay; genistein (200 µM), wortmannin (20 nM), cytochalasin D (50 µM), PP2 (10 µM) and methyl-β-cyclodextrin (2 mM).

### Construction of FnBPA variants

FnBPA constructs containing various combinations of Fn-binding repeats were produced using a circular PCR system based on that described by Massey et al. [19]. Primers were designed to various repeats or DNA just outside of the *fnb*A repeat region and were synthesized with 5′ phosphorylation in order to allow self-ligation of the PCR product. Primer sequences and the combinations used to produce the various products can be found in Table 1. Plasmid pFnBA4 [34] was used as a template for the amplification of DNA lacking in various repeats. PCR was performed using Phusion high-fidelity DNA polymerase (New England Biolabs (NEB) Ipswich, MA). Template DNA was digested with *Dpn*I and products cleaned using a PCR purification kit or gel extracted (Qiagen, Hilden, Germany). Linear DNA was self-ligated using T4 DNA ligase (NEB) to produce plasmids containing DNA encoding the *fnb*A gene lacking various combinations of FnBRs (Table 2). Ligated PCR products were transformed into CaCl_2_ competent *E. coli* [43] and plated onto LB agar containing 100 µg ml^−1^ ampicillin. Selected colonies were grown overnight in LB broth (5 ml, 100 µg ml^−1^ Ampicillin), bacteria pelleted by centrifugation and plasmids recovered using a miniprep kit (Qiagen). Constructs were checked by sequencing (Geneservice, London, UK) and transformed into *S. aureus* RN4220 by electroporation. Plasmids were recovered using a combination of lysostaphin and the Qiagen miniprep kit [19] and re-transformed into *S. aureus* 8325.4 Δ*fnb*.

### Construction and controlled expression of FnBPA variants in *L. lactis*

Controlled expression of FnBPA variants was achieved using a nisin-inducible system previously used to control the expression of the enterococcal surface protein PrgB [35]. The *prg*B gene was excised from plasmid pMSP7517 using *Nco*I and *Xho*I and the remaining plasmid recovered by gel excision. pFnBA4 and associated constructs were used as templates for PCR reactions using primers designed to amplify the entire *fnb*A gene (and repeat-region variants) and contained *Nco*I (AAACCATGGAGGAGGTATTATAGTGAAAAACAATCTTAGG) and *Xho*I (AAACTCGAGCTAACTTTATCTCTCAGTTCGTTATC) sites respectively (underlined). Digested PCR products were ligated into digested pMSP7517 and transformed into CaCl_2_-treated *E. coli* K12 ER2925, which was more suitable for antibiotic selection using erythomycin than *E. coli* DH5α (data not shown). Plasmids were recovered, checked by sequencing and electro-transformed into *L. lactis* NZ9800 [36]. Expression was induced by culture (16 h, 30°C) of *L. lactis* containing FnBPA variant constructs (Table 2) in the presence of various concentrations of nisin (range 0–200 ng ml^−1^). Expression levels were determined by ELISA and binding to immobilized fibrinogen (see below).

### Determination of surface protein expression by Western immunoblot

*S. aureus* was cultured as described and the bacteria pelleted by centrifugation (5,000 x g, 8 min). Bacteria were washed 3 times in PBS, resuspended in spheroplasting buffer (30% raffinose, Tris-HCl pH 7.5) [27] containing 100 µg ml^−1^ lysostaphin and incubated for 1 h at 37°C. Spheroplasts were pelleted by centrifugation and the supernatant containing surface proteins recovered. Aliquots (40 µl) were mixed with 5X concentrated sample buffer (10 µl; 50% glycerol containing 50 mM Tris-HCl pH 6.8, 10% SDS and 10 mM 2-mercaptoethanol) and heated at 100°C for 5 min before being subjected to SDS-PAGE (7.5% acrylamide). Separated proteins were electroblotted onto nitrocellulose membrane using a BioRad (Hercules, Ca) semi-dry blotter (25V, 1 h).

Membranes were blocked by incubation in 3% BSA (1 h) and probed with antibodies to the N-terminal domain of FnBPA [19] for 1 h with agitation. Membranes were washed 4 times with PBS and incubated with HRP-conjugated goat anti-rabbit antibodies (1 h with agitation). Membranes were subsequently washed 4 times with PBS, rinsed with ddH_2_O and reactive bands detected using the Opti-4CN detection kit (BioRad).

### Determination of surface protein expression by ELISA

*S. aureus* strains were cultured as described above, washed with PBS, adjusted to OD_600_ = 0.1 and aliquots (50 µl) immobilized onto triplicate plastic wells (Nunc Maxisorp) by incubation at 4°C for 16 h. Wells were blocked with BSA (1 h, 4°C), washed once with PBS and then incubated with antibodies (50 µl, 1∶500 in PBS for *S. aureus* 8325.4 or 1∶2000 for *S. aureus* LS-1) raised to the A region of FnBPA [19] for 1 h at 37°C. Wells were washed 4 times with PBS and incubated with HRP-conjugated protein A (50 µl, 1∶5000, Sigma) for 30 min at 37°C. Wells were washed a further 4 times with PBS and bound antibody detected and quantified with 100 µl TMB (3,3′,5,5′-tetramethylbenzidine) ELISA detection substrate (Sigma) for 10 min at room temperature. The reaction was stopped with 100 µl 2M HCl and product measured at 450 nm using a microplate reader.

*L. lactis* expressing FnBPA variants were washed in PBS as described above, diluted 1∶16 in PBS and 50 µl aliquots added to duplicate wells of a microtitre plate as described for *S. aureus*. Wells were blocked as described above and immobilized bacteria were incubated with anti-FnBPA antibodies (50 µl, 1∶1000, 1 h, 37°C) before washing three times with PBS. Bacteria were then incubated with HRP-conjugated anti-rabbit antibodies (50 µl, 1∶5000, 1 h, 37°C) and quantified using TMB as described above.

### Solid-phase Fn and fibrinogen binding assay

The adhesion of bacteria to human Fn (Sigma), murine Fn (Biopur, Bubendorf, Switzerland) or fibrinogen (Fn-depleted, Enzyme Research Labs, Swansea, UK) was assessed using a protocol based on those of Jakubovics et al. and Peacock et al. [27], [44]. Fn or fibrinogen (1 µg protein in 100 µl PBS [pH 7.4] per well) was immobilized onto plastic Nunc Maxisorp Immuno modules by incubation at 4°C for 16 h and remaining binding sites were blocked with 300 µl 3% BSA (in PBS) at 25°C for 1 h. Blocked wells were washed with PBS and incubated with 100 µl bacteria (approx. 1×10^8^ *S. aureus* or 5×10^8^ *L. lactis* in PBS) at 37°C for 1 h. Non-adherent bacteria were removed by 3 rounds of washing with PBS and adherent bacteria fixed with 0.25% paraformaldehyde (5 min, 25°C). Wells were washed with PBS a further 2 times. Fixed bacteria were stained with crystal violet (0.5%, 2 min) before a further three rounds of washing, as described above. Adherent, fixed bacteria were enumerated by solubilization of crystal violet with 100 µl 7% acetic acid. The contents of each well was quantified by measurement at A_595_ using a microplate reader. For both Fn and fibrinogen binding, readings were blanked against bacteria binding to wells coated only with BSA. Absorbance measurements were converted to bacterial numbers by the use of standard plots of known bacterial numbers against A_595_ readings (Figure S3). These showed that the relationship of A_595_ to bacterial numbers is linear (Figure S3). Each experiment was performed at least three times. Since *S. aureus* was stained more strongly with crystal violet than *L. lactis* (Figure S3), different amounts of bacteria were used in assays in order to allow bacterial quantification over as wide a range of adhesion levels as possible.

### Endothelial adhesion and invasion assays

Cultured cells were dissociated from plastic flasks using trypsin-EDTA solution (Invitrogen) and approximately 5×10^5^ (in 0.5 ml medium) were seeded into each well of 24-well plates (Nunc) containing 13 mm plastic Thermanox cover slips (Fisher) and allowed to attach for 48 h (37°C, 5% CO_2_). Coverslips were dip-washed once in PBS and placed in the well of a new 24-well plate containing 450 µl of DMEM containing 10% FBS.

To each well, 50 µl of washed bacteria were added (approximately 1×10^7^ CFU *S. aureus* or 5×10^7^ *L. lactis*) and incubated for 5–90 minutes at 37°C in 5% CO_2_.

To measure the total number of bacteria associated with the cells (adherent and internalized), coverslips were dip-washed 3 times in PBS and added to wells containing 500 µl 0.5% Triton X-100. Wells containing coverslips were agitated by pipetting to fully lyse the cells and CFU were enumerated by serial dilution and plating onto TSA agar plates and incubated overnight at 37°C in 5% CO_2_.

For invasion assays, the bacterial suspension was removed and replaced with 500 µl DMEM/10% FBS supplemented with 200 µg ml^−1^ gentamicin and incubated at 37°C in 5% CO_2_ for 60 min. Coverslips were washed 3 times in PBS, lysed and plated for CFU as described for the adhesion assay above. In assays where metabolic inhibitors were used, these were added to cell monolayers for 60 min prior to the experiment and concentrations maintained during incubation with bacteria.

### Statistics

For adhesion and invasion assays, statistical analyses were performed with Student's *t* test by using the Bonferroni correction for multiple comparisons. Values that were statistically significantly different from control values are indicated by asterices in the figures. Error bars indicate the mean average ± standard deviation of multiple independent experiments (indicated in the figure legend).

### Whole human blood model of infection

The ability of *S. aureus* to survive in human blood and generate pro-inflammatory cytokine release was assessed using a whole human blood model. Blood from a healthy male was drawn into heparinised tubes and used immediately. Bacteria were washed and diluted in PBS and 10 µl (containing approximately 20,000 CFU) was added to 800 µl blood and incubated at 37°C for 1, 2 or 6 h with mixing. Aliquots of the blood/bacteria mixture (5 µl) were mixed with PBS (95 µl) and plated onto tryptic soy agar to enumerate CFU. In addition to plating for CFU, blood at the final time point (6 h) was centrifuged (5,000 x *g*, 5 min) and 100 µl serum recovered for analysis of TNFα production using a BD OptEIA ELISA kit (BD Biosciences, San Diego, USA).

### Murine sepsis model

Groups of 15 mice were infected by intravenous injection with *S. aureus* strain LS-1 Δ*fnb* [14] expressing FnBPR1-11 (1.1×10^7^ CFU), FnBPR0 (1.1×10^7^ CFU), FnBPR1 (1.0×10^7^ CFU) or FnBPR1,10,11 (1.2×10^7^ CFU) and mortality and weight change were followed until day 14. The clinical evaluation was performed in a blinded manner. The overall condition of each mouse was examined by assessing signs of systemic inflammation, i.e. weight decrease, reduced alertness, and ruffled coat. In keeping with approved husbandry standards, in cases of severe systemic infection, when a mouse was judged too ill to survive another 24 h, it was killed by cervical dislocation and considered dead due to sepsis. The bacterial load in kidneys was assessed as described previously [14]. Two independent groups of 4 mice were inoculated with *S. aureus* (as described above) and sacrificed 3 days later. Kidney pairs were recovered and CFU enumerated on TSA plates. Retention of the FnBPA-encoding plasmid was determined by plating onto TSA with and without chloramphenicol. Statistical evaluation was done by using the Kruskal-Wallis test with a following post-hoc analysis, or the Logrank (Mantel-Cox) test at survival analysis. P = <0.05 was considered to be significant. No correction for multiple comparisons was employed. Data are reported as medians, interquartile ranges, and 80% central ranges, unless otherwise mentioned.

### Gene accession information

FnBPA variants were derived from the *fnb*A gene of *S. aureus* 8325.4, Swissprot accession SAOUHSC_02803.

## Supporting Information

Figure S1 FnBPA expression ELISA standards. The reactivity of anti-N-terminal FnBPA antibodies with *S. aureus* or *L. lactis* was measured by ELISA over a range of two-fold dilutions from a starting point of OD_600_  = 1.0. A, B. Reactivity of anti-N-terminal FnBPA antibodies with *S. aureus* 8325.4 (A) or LS-1 (B). Open circles represent values obtained with *S. aureus* pFnBPR0. Open squares represent values obtained with *S. aureus* Δ*fnb*. These were used to produce blanked values (filled triangles) that took into account disruption caused by protein A. C. Reactivity of anti-N-terminal FnBPA antibodies with *L. lactis* expressing FnBPR1-11 (induced with 10 ng ml^−1^ nisin).
(0.12 MB TIF)

Figure S2 The interaction of *S. aureus* with EA. hy926 cells is similar to HUVECs. A. Adhesion to and entry into EA. hy926 cells and HUVECs by *S. aureus* 8325.4 over time. B. Bacterial uptake by EA. hy926 cells and HUVECs of *S. aureus* 8325-4 (WT), isogenic mutants deficient in Spa (Δ*spa*) or both *fnb*A and *fnb*B (Δ*fnb*) and the *fnb*-defective strain complemented with a plasmid expressing full-length *fnb*A (Comp). C. Bacterial uptake by EA. hy926 cells and HUVECs of *L. lactis* expressing *fnb*A (FnBPA+) or containing an empty control plasmid (CTL). D. The uptake of *S. aureus* 8325.4 by EA. hy926 cells and HUVECs treated with the metabolic inhibitors genistein (GEN), methyl-β-cyclodextrin (MCD), cycloheximide (CHX), wortmannin (WRT), cytochalasin D (CD), filipin (FIL) or PP2. Untreated cells are labelled CTL. Values that differed significantly (ttest) from untreated cells are marked (*). Experiments were performed 3 times in duplicate wells.
(1.91 MB TIF)

Figure S3 Standard plots showing the linear relationship between bacterial numbers and A_595_ when using crystal violet to quantify bacterial adhesion.
(0.10 MB TIF)
