# Supplementary figures and images for: Staphylococcus aureus Host Cell Invasion and Virulence in Sepsis Is Facilitated by the Multiple Repeats within FnBPA
Source: PLoS Pathog. 2010 Jun 24;6(6):e1000964. doi: 10.1371/journal.ppat.1000964 (PMC2891841; doi:10.1371/journal.ppat.1000964)

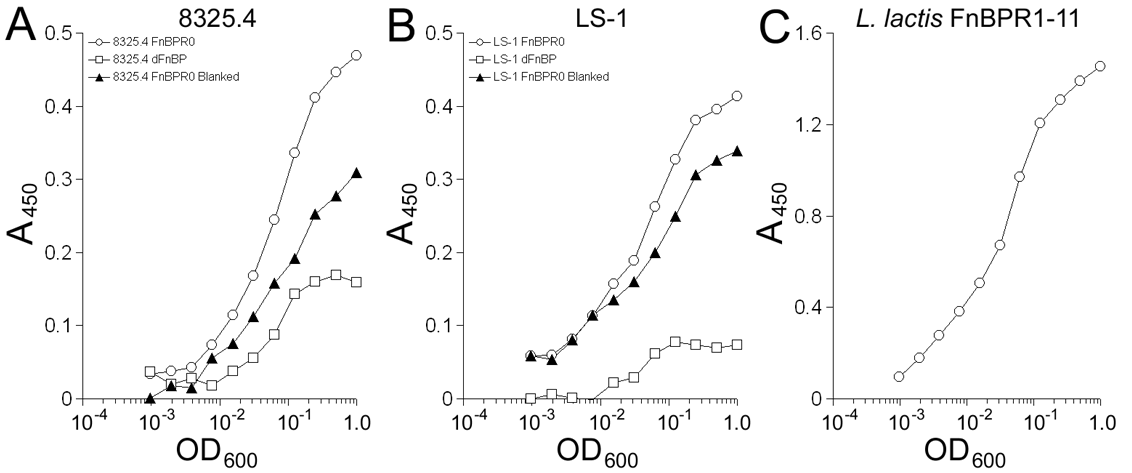

Supplement: Figure S1 — FnBPA expression ELISA standards. The reactivity of anti-N-terminal FnBPA antibodies with S. aureus or L. lactis was measured by ELISA over a range of two-fold dilutions from a starting point of OD600 = 1.0. A, B. Reactivity of anti-N-terminal FnBPA antibodies with S. aureus 8325.4 (A) or LS-1 (B). Open circles represent values obtained with S. aureus pFnBPR0. Open squares represent values obtained with S. aureus Δfnb. These were used to produce blanked values (filled triangles) that took into account disruption caused by protein A. C. Reactivity of anti-N-terminal FnBPA antibodies with L. lactis expressing FnBPR1-11 (induced with 10 ng ml−1 nisin). (0.12 MB TIF) [file ppat.1000964.s001.tif]

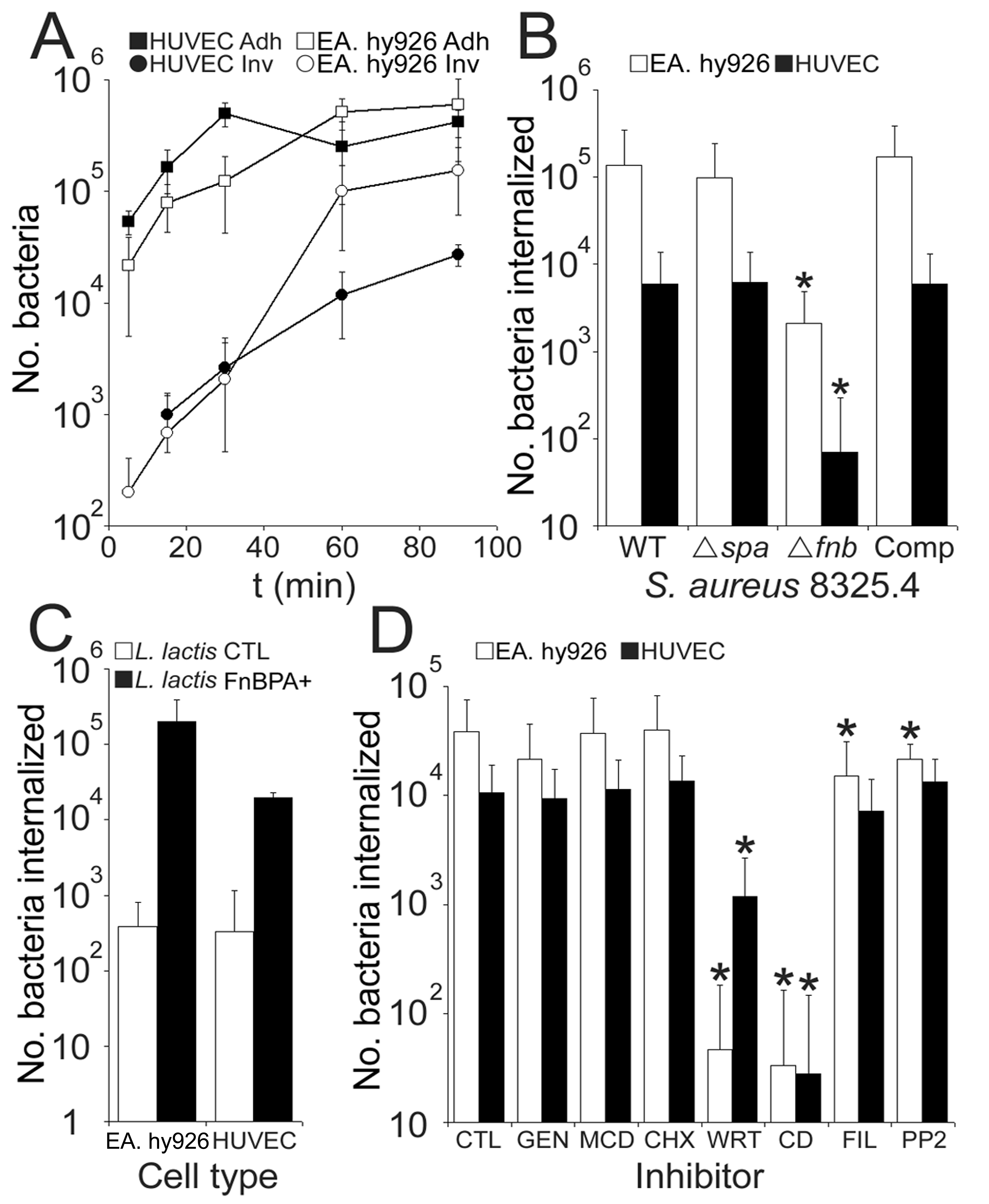

Supplement: Figure S2 — The interaction of S. aureus with EA. hy926 cells is similar to HUVECs. A. Adhesion to and entry into EA. hy926 cells and HUVECs by S. aureus 8325.4 over time. B. Bacterial uptake by EA. hy926 cells and HUVECs of S. aureus 8325-4 (WT), isogenic mutants deficient in Spa (Δspa) or both fnbA and fnbB (Δfnb) and the fnb-defective strain complemented with a plasmid expressing full-length fnbA (Comp). C. Bacterial uptake by EA. hy926 cells and HUVECs of L. lactis expressing fnbA (FnBPA+) or containing an empty control plasmid (CTL). D. The uptake of S. aureus 8325.4 by EA. hy926 cells and HUVECs treated with the metabolic inhibitors genistein (GEN), methyl-β-cyclodextrin (MCD), cycloheximide (CHX), wortmannin (WRT), cytochalasin D (CD), filipin (FIL) or PP2. Untreated cells are labelled CTL. Values that differed significantly (ttest) from untreated cells are marked (*). Experiments were performed 3 times in duplicate wells. (1.91 MB TIF) [file ppat.1000964.s002.tif]

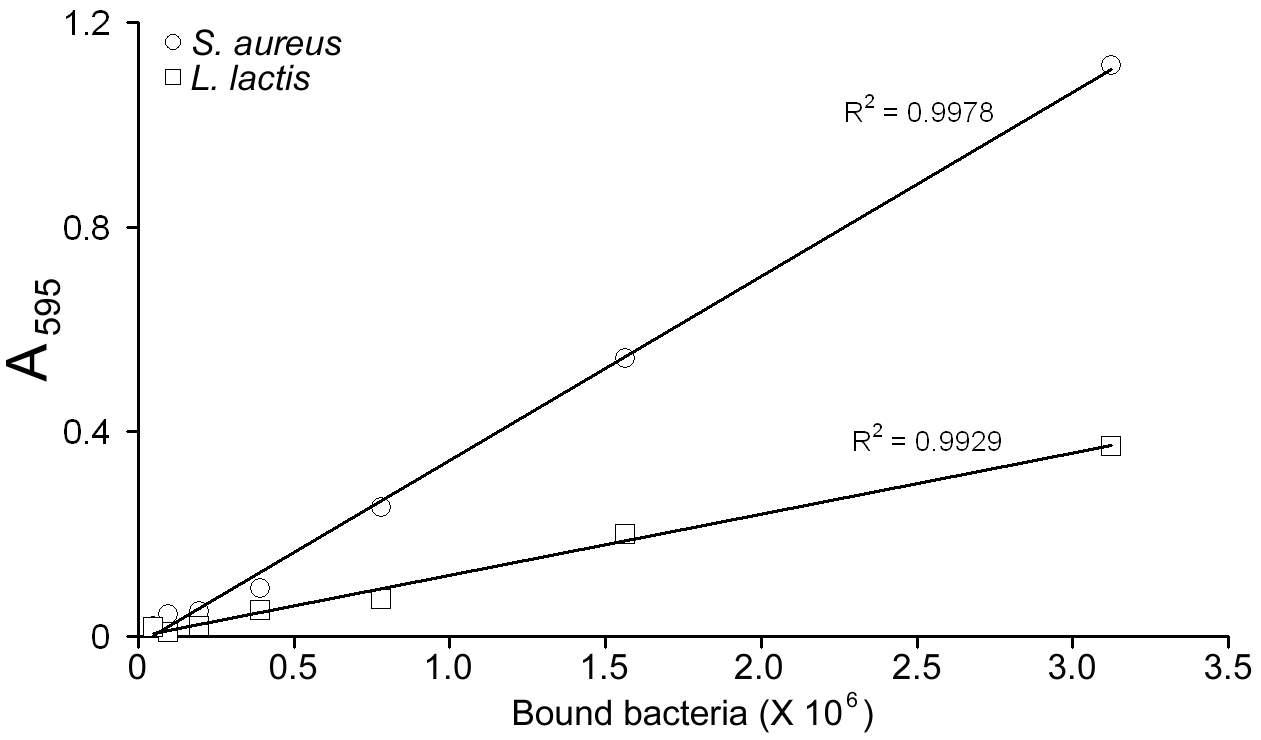

Supplement: Figure S3 — Standard plots showing the linear relationship between bacterial numbers and A595 when using crystal violet to quantify bacterial adhesion. (0.10 MB TIF) [file ppat.1000964.s003.tif]
